# Supplementary figures and images for: The Capsule Regulatory Network of Klebsiella pneumoniae Defined by density-TraDISort
Source: mBio. 2018 Nov 20;9(6):e01863-18. doi: 10.1128/mBio.01863-18 (PMC6247091; doi:10.1128/mBio.01863-18)

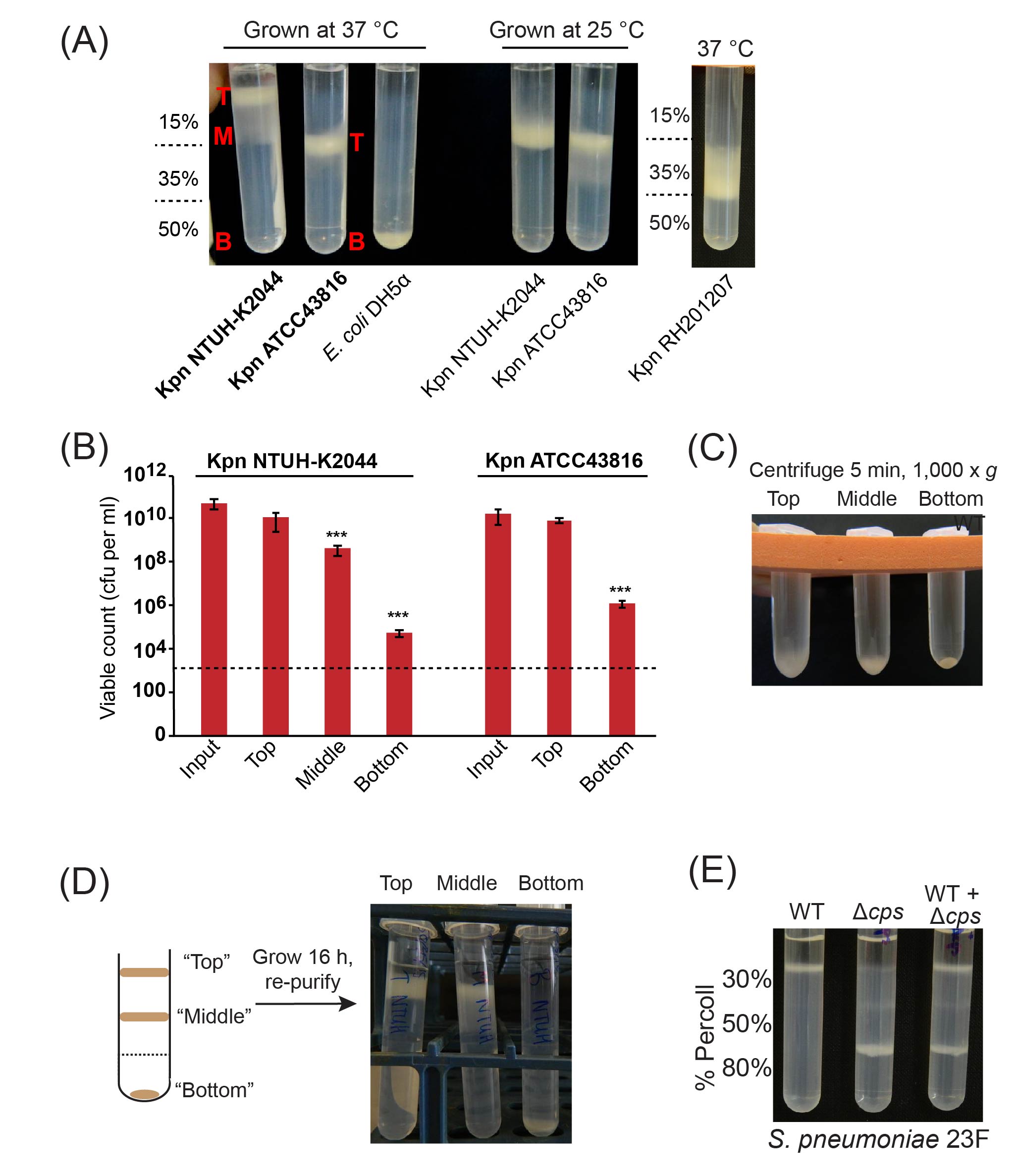

Supplement: FIG S1 [file mbo006184168sf1.jpg]

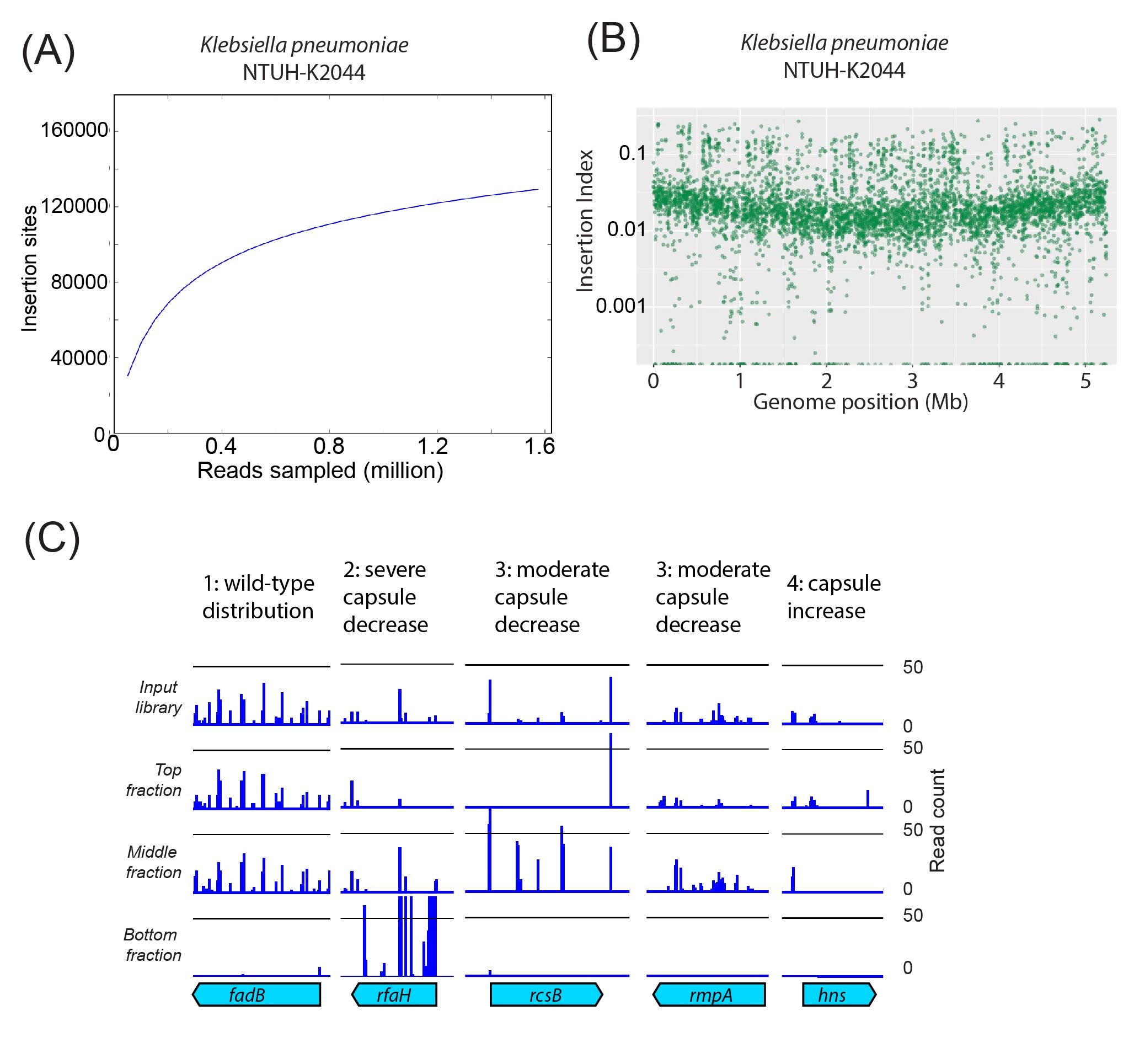

Supplement: FIG S2 [file mbo006184168sf2.jpg]

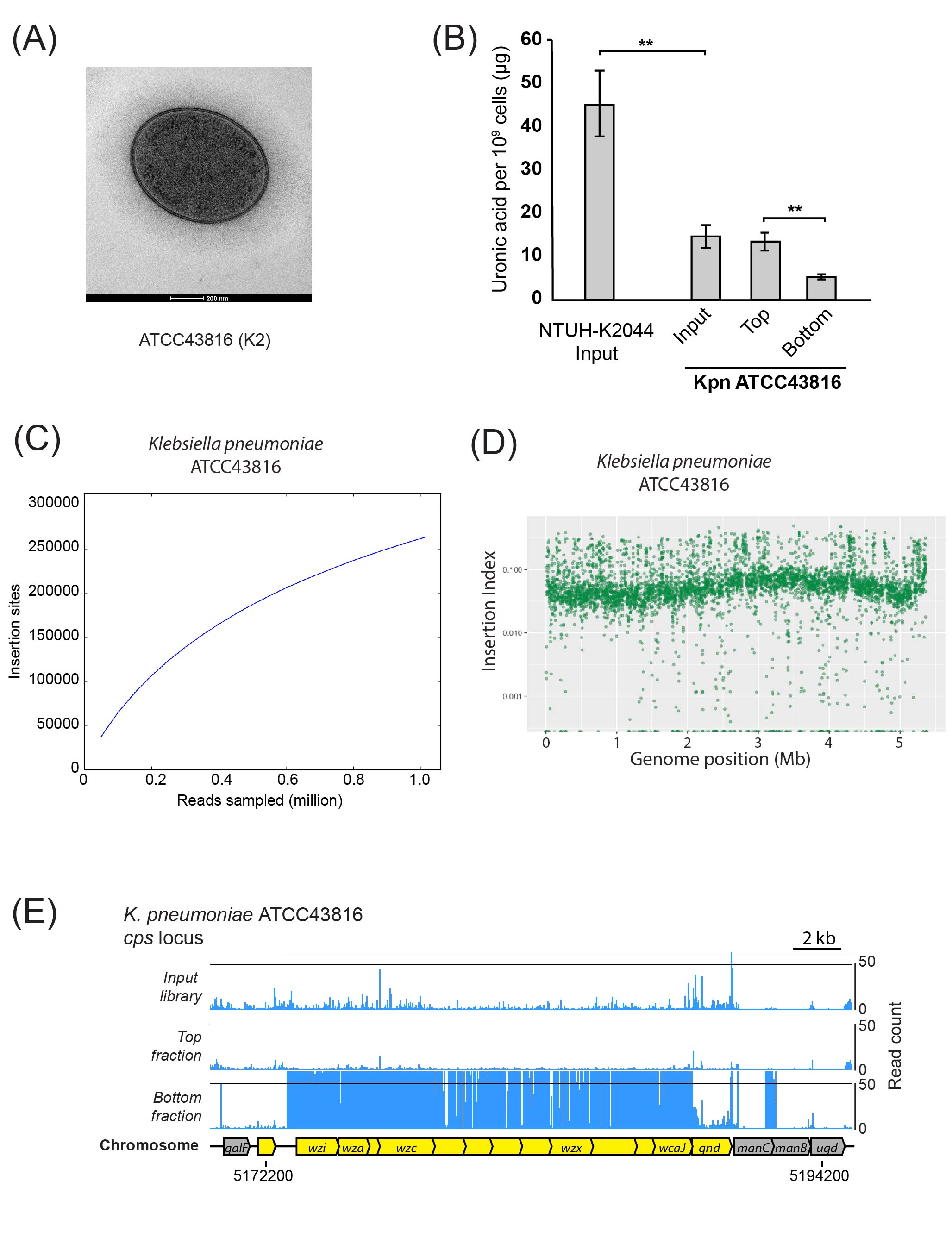

Supplement: FIG S3 [file mbo006184168sf3.jpg]

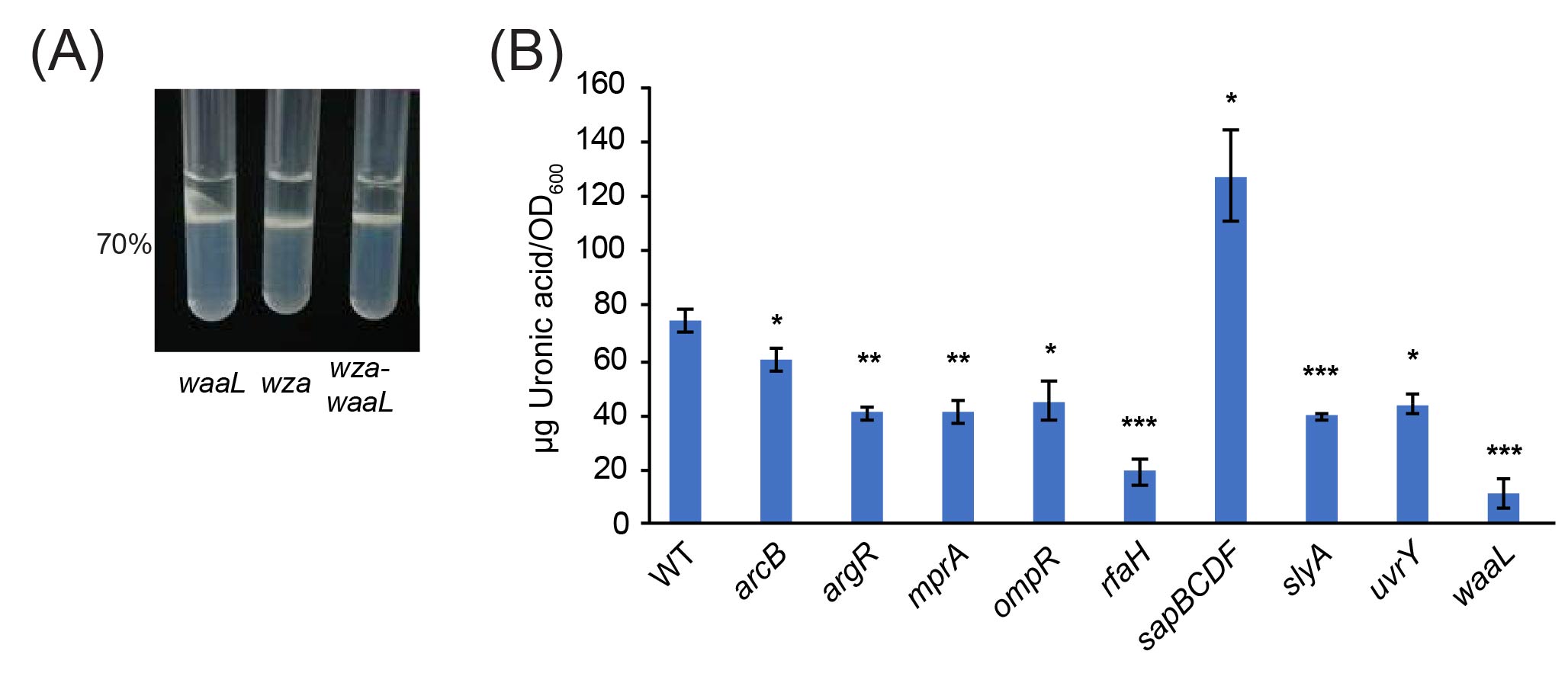

Supplement: FIG S4 [file mbo006184168sf4.jpg]
