# Supplementary material for: The Capsule Regulatory Network of Klebsiella pneumoniae Defined by density-TraDISort
Source: mBio. 2018 Nov 20;9(6):e01863-18. doi: 10.1128/mBio.01863-18 (PMC6247091; doi:10.1128/mBio.01863-18)
Supplement: TEXT S1 [file mbo006184168s1.docx]

**Extended methods**

*Generation of transposon insertion libraries*

TraDIS libraries were generated using the transposon delivery plasmid pDS1028 (1), which contains a Tn*5*-based transposon comprising an *ori6K* replication origin, chloramphenicol acetyltransferase gene, and the Ω transcriptional and translational terminator flanked by Tn*5* inverted repeats. The pDS1028 plasmid was introduced to recipient *Klebsiella* by conjugation. The donor strain *Escherichia coli* β2163 pDS1028 was grown overnight at 37 ˚C on LB agar supplemented with 150 μM 2-6-diaminopimelic acid (DAPA) and 12.5 μg/ml tetracycline. Recipient *Klebsiella* strains were grown overnight on LB agar at 25 ˚C. Donor and recipient strains were resuspended in PBS and adjusted to an OD_600_ of 40 and 20, respectively. Multiple conjugation patches of 25 μl donor + 25 μl recipient were set up on LB agar plates containing 150 μM DAPA, dried, and incubated at 37 ˚C for 1 h. Mating patches were then suspended in 50% glycerol and flash-frozen.

Aliquots of the conjugation mixture were thawed and plated on LB agar with and without chloramphenicol to determine the viable count of transposon-containing *K. pneumoniae*. For the *K. pneumoniae* ATCC 43816 library, bioassay dishes of LB supplemented with 15 μg/ml chloramphenicol were seeded with 10,000 transposon mutants and incubated overnight at 37 ˚C. A total of ~700,000 transposon mutant colonies were resuspended in sterile 25% glycerol 0.5 x PBS and flash-frozen. The *K. pneumoniae* NTUH-K2044 conjugation was seeded at a lower density as this was determined to be necessary for robust selection of mutants; for this library, LB + 37.5 μg/ml chloramphenicol bioassay plates were spread with 3,000 - 4,000 transposon mutants and incubated overnight at 25 ˚C (this was necessary to avoid the excess capsule production observed when grown at 37 ˚C). A total of ~250,000 transposon mutant colonies were resuspended in 25% glycerol 0.5 x PBS and flash-frozen. At least 20 individual colonies from each library were tested by PCR to confirm the presence of the transposon and the loss of the pDS1028 backbone, and the loss of the vector and counterselection of the donor strain in the TraDIS mutant library was also confirmed by qRT-PCR.

*Mutant library fractionation on Percoll gradients*

Bacterial mutant libraries were separated on the basis of their capsule expression by sedimentation on a Percoll (GE Healthcare) density gradient (Fig. 1B). All separations and subsequent DNA isolations were performed in biological triplicate. Bacteria were collected from overnight cultures (10 ml) by centrifugation and were resuspended in 1 ml 1 X PBS. The suspension (600 μl) was applied to the top of a density gradient of various concentrations of Percoll in 1 x PBS; 50%, 35% and 15%, to resolve NTUH-K2044 fractions, and 50% and 35%, to resolve ATCC 43816 fractions. The remaining bacterial suspension was retained and used as the ‘input’ fraction in subsequent steps. Percoll (GE) was diluted in PBS to achieve the desired concentrations, and the discontinuous gradient was set up in 6 ml blood tubes (Greiner Bio-one, 456238) from bottom to top using a 200 μl pipette. Cells were fractionated in a fixed angle rotor (Eppendorf F-34-638) for 30 min at 3,000 x *g* (room temperature). Resultant fractions were recovered by pipetting, washed in 1 ml PBS to remove residual Percoll, and resuspended in 1 ml PBS. Fractions which were not visibly turbid upon resuspension were allowed to outgrow for two hours in 10 ml LB (37 ˚C, 180 rpm). Outgrown cells were collected by centrifugation, washed, and resuspended as before.

Random-prime PCR of selected single mutants was performed as described (2) using primers FS57-59 and FS109 and Herculase II polymerase (Agilent). Amplicons were sequenced using primer FS107.

*DNA extraction and next-generation sequencing*

Genomic DNA (gDNA) was prepared from each Percoll-resolved fraction by phenol-chloroform extraction. From each gDNA preparation, 2 μg DNA was used to prepare TraDIS transposon-specific sequencing libraries as described previously, using primer FS108 for specific amplification of transposon junctions (3). Sequencing was carried out on the Illumina MiSeq platform using primer FS107.

*Analysis of TraDIS data*

At least 900,000 transposon-containing, uniquely-mapped reads were obtained per sample. These insertion sites were distributed relatively evenly across the genome in both strains (Fig. S2B and S3D), and a rarefaction curve of reads and insertion sites in the input library fraction showed that the NTUH-K2044 library was sequenced to near-saturation, while greater sequencing depth would increase the number of mapped insertion sites in the ATCC 43816 library.

The analysis of TraDIS sequencing results was carried out as described previously (3, 4), with some modifications. Briefly, transposon tag reads were added to sequencing reads, and the resulting reads mapped to the relevant reference genome, using the add_tradis_tags and bacteria_tradis scripts by Barquist *et al*. (3). The following parameters were passed to bacteria_tradis: "-v --smalt_r -1 --smalt_y 0.98 -t TAAGAGACAG -mm 1". The triplicate plot files produced for each condition were analysed using a customised Python script to count gene-wise transposon insertion sites, with reads mapping to the 3' 10% of the gene discarded (available at <https://github/francesca-short/tradis_scripts>). Different fractions were compared to the input sample using tradis_comparison.R in all cases. Reduced capsule hits were defined as genes with reduced mutant abundance in the top fraction (log_2_FC < -1, q-value <0.001) and increased abundance in the bottom or middle fraction (log_2_FC > 1, q-value < 0.001). Increased capsule hits were defined as those with dramatically reduced mutant abundance in the middle fraction (log_2_FC < -3; q-value < 0.001) without concomitant enrichment in the bottom fraction (log_2_FC < 1), with genes showing very few reads in any fraction excluded (*i.e.*, log_2_(counts per million) in the top fraction was greater than 4).

Enrichment analysis was conducted using the topGO R package (5). GO terms were assigned to protein-coding sequences using eggNOG-Mapper at <http://eggnogdb.embl.de> (6) based on eggNOG 4.5 orthology data (7), using experimental only terms and DIAMOND mapping.

*K. pneumoniae* genome assemblies annotated using Prokka (8, 9) were used to generate a pan-genome using Roary (10) with the following options: "-e -i 90". Included in this genome set were Prokka-annotated sequences for the two *K. pneumoniae* strains used in this study, and GFF-formatted copies of their respective annotated reference sequences.

*Quantification of capsule by uronic acid assay*

The amount of capsule elaborated by *K. pneumoniae* was quantified using an assay for uronic acid described previously (11, 12), with modifications. To extract capsule from *K. pneumoniae* gradient fractions, overnight cultures were fractionated on a Percoll gradient and recovered as described above. Cells were then pelleted, resuspended in PBS to an OD_600_ of 4, and 0.5 ml of this suspension was used for capsule extraction. To extract capsule from different *K. pneumoniae* mutant strains, 0.5 ml aliquots of overnight culture (5 ml LB, 37 °C) were used directly without exchange of media. Capsule extraction buffer (100 μl; 500 mM citric acid pH 2.0, 1% Zwittergent 3-10) was added to the suspension, and the mixtures were incubated at 50 ˚C for 20 min before centrifugation to pellet cellular debris (5 min, 18,000 x *g*, room temperature). Capsule components were precipitated by incubating aliquots of supernatant (300 μl) with 1.5 ml absolute ethanol at 4 ˚C for 30 min. The precipitates were collected by centrifugation (20 min, 18,000 x *g*, 4 ˚C), air-dried, and resuspended in 200 μl sterile water. The amount of capsule present was quantified by incubating 100 μl capsule suspension with 0.6 ml tetraborate solution (12.5 mM disodium tetraborate in sulfuric acid) on ice for 10 min, followed by incubation at 95 ˚C for 5 min, and immediate cooling on ice for at least 5 min. The absorbance of the sample at 520 nm was measured before and after the addition of hydroxyphenyl reagent (10 μl, 3-hydroxybiphenyl in 0.5% sodium hydroxide). A standard curve using glucuronic acid (Sigma-Aldrich) was used to calculate uronic acid concentrations.

**Random-prime PCR identification of transposon insertion sites in acapsular clones**

| **Clone** | **Location** | **Strand** | **Feature** | **Feature orientation** |
| --- | --- | --- | --- | --- |
| 1 | 3541246 | F | *wza* | R |
| 2 | 4345859 | F | *togM* | F |
| 3 | 3541546 | F | *wza* | R |
| 4 | 3542681 | F | *wzi* | R |
| 5 | 201786 | F | *rfaH* promoter | R |
| 6 | 3541293 | F | *wza* | R |
| 7 | 3525235 | R | *manC* | R |

**Supplementary references**

1. Monson R, Smith DS, Matilla MA, Roberts K, Richardson E, Drew A, Williamson N, Ramsay J, Welch M, Salmond GPC. 2015. A plasmid-transposon hybrid mutagenesis system effective in a broad range of enterobacteria. Front Microbiol 6:1–13.

2. Fineran PC, Everson L, Slater H, Salmond GPC. 2005. A GntR family transcriptional regulator (PigT) controls gluconate-mediated repression and defines a new, independent pathway for regulation of the tripyrrole antibiotic, prodigiosin, in *Serratia*. Microbiology 151:3833–3845.

3. Barquist L, Mayho M, Cummins C, Cain AK, Boinett CJ, Page AJ, Langridge GC, Quail MA, Keane JA, Parkhill J. 2016. The TraDIS toolkit: Sequencing and analysis for dense transposon mutant libraries. Bioinformatics 32:1109–1111.

4. Langridge GC, Phan MD, Turner DJ, Perkins TT, Parts L, Haase J, Charles I, Maskell DJ, Peters SE, Dougan G, Wain J, Parkhill J, Turner a. K. 2009. Simultaneous assay of every *Salmonella* Typhi gene using one million transposon mutants. Genome Res 19:2308–2316.

5. Alexa A, Rahnenfuhrer J. 2016. topGO: Enrichment Analysis for Gene Ontology.

6. Huerta-Cepas J, Forslund K, Coelho LP, Szklarczyk D, Jensen LJ, von Mering C, Bork P. 2017. Fast genome-wide functional annotation through orthology assignment by eggNOG-Mapper. Mol Biol Evol 34:2115–2122.

7. Huerta-Cepas J, Szklarczyk D, Forslund K, Cook H, Heller D, Walter MC, Rattei T, Mende DR, Sunagawa S, Kuhn M, Jensen LJ, von Mering C, Bork P. 2016. eggNOG 4.5: a hierarchical orthology framework with improved functional annotations for eukaryotic, prokaryotic and viral sequences. Nucleic Acids Res 44:D286–D293.

8. Page AJ, De Silva N, Hunt M, Quail MA, Parkhill J, Harris SR, Otto TD, Keane JA. 2016. Robust high-throughput prokaryote de novo assembly and improvement pipeline for Illumina data. Microb Genomics 2.

9. Seemann T. 2014. Prokka: rapid prokaryotic genome annotation. Bioinformatics 30:2068–2069.

10. Page AJ, Cummins CA, Hunt M, Wong VK, Reuter S, Holden MTG, Fookes M, Falush D, Keane JA, Parkhill J. 2015. Roary: Rapid large-scale prokaryote pan genome analysis. Bioinformatics 31:3691–3693.

11. Favre-Bonté S, Licht TR, Forestier C, Krogfelt KA. 1999. *Klebsiella pneumoniae* capsule expression is necessary for colonization of large intestines of streptomycin-treated mice. Infect Immun 67:6152–6156.

12. Lawlor MS, Hsu J, Rick PD, Miller VL. 2005. Identification of *Klebsiella pneumoniae* virulence determinants using an intranasal infection model. Mol Microbiol 58:1054–1073.

13. Broberg CA, Wu W, Cavalcoli JD, Miller VL, Bachman MA. 2014. Complete genome sequence of *Klebsiella pneumoniae* strain ATCC 43816 KPPR1, a rifampin-resistant mutant commonly used in animal, genetic, and molecular biology studies. Genome Announc 2.

14. Wu KM, Li NH, Yan JJ, Tsao N, Liao TL, Tsai HC, Fung CP, Chen HJ, Liu YM, Wang JT, Fang CT, Chang SC, Shu HY, Liu TT, Chen YT, Shiau YR, Lauderdale TL, Su IJ, Kirby R, Tsai SF. 2009. Genome sequencing and comparative analysis of *Klebsiella pneumoniae* NTUH-K2044, a strain causing liver abscess and meningitis. J Bacteriol 191:4492–4501.

15. Demarre G, Guérout AM, Matsumoto-Mashimo C, Rowe-Magnus DA, Marlière P, Mazel D. 2005. A new family of mobilizable suicide plasmids based on broad host range R388 plasmid (IncW) and RP4 plasmid (IncPα) conjugative machineries and their cognate *Escherichia coli* host strains. Res Microbiol 156:245–255.

16. Poulter S. 2011. PhD thesis. Department of Biochemistry, University of Cambridge. The LuxR-family quorum sensing transcriptional regulator CarR in *Erwinia* and *Serratia*.
