# Supplementary material for: The Capsule Regulatory Network of Klebsiella pneumoniae Defined by density-TraDISort
Source: mBio. 2018 Nov 20;9(6):e01863-18. doi: 10.1128/mBio.01863-18 (PMC6247091; doi:10.1128/mBio.01863-18)
Supplement: TABLE S1 [file mbo006184168st1.docx]

**Supplementary Table S1**. Strains, plasmids, and oligonucleotides used in this study. Tc^R^ = tetracycline resistant; Cm^R^ = chloramphenicol resistant.

| **Bacterial strains** | | | | | | |
| --- | --- | --- | --- | --- | --- | --- |
| **Strain** | | **Description/genotype** | | **Source** | | |
| *Klebsiella pneumoniae* ATCC 43816 | | Mouse-virulent sequence type 493, K-type 2, O-antigen type 1 | | American Type Culture Collection; (13) | | |
| *Klebsiella pneumoniae* NTUH-K2044 | | Hypervirulent, hypermucoid liver abscess isolate, sequence type 23, K-type 1, O-antigen type 1 | | Jin-Town Wang, National Taiwan University Hospital; (14) | | |
| *Escherichia coli* β2163 | | F^-^ RP4-2-Tc::Mu Δ*dapA*::*(erm-pir)* | | (15) | | |
| *Streptococcus* *pneumoniae* 23F ATCC700669 | |  | |  | | |
| *Streptococcus* *pneumoniae* 23F ATCC700669 | | Δ*cps* | |  | | |
| *K. pneumoniae* ATCC43816 | | Δ*arnF* | | This study | | |
| *K. pneumoniae* ATCC43816 | | Δ*emrR* | | This study | | |
| *K. pneumoniae* ATCC43816 | | Δ*rfaH* | | This study | | |
| *K. pneumoniae* NTUH-K2044 | | Δ*arcB* | | This study | | |
| *K. pneumoniae* NTUH-K2044 | | Δ*argR* | | This study | | |
| *K. pneumoniae* NTUH-K2044 | | Δ*emrR* | | This study | | |
| *K. pneumoniae* NTUH-K2044 | | Δ*ompR* | | This study | | |
| *K. pneumoniae* NTUH-K2044 | | Δ*rfaH* | | This study | | |
| *K. pneumoniae* NTUH-K2044 | | Δ*sapBCDF* | | This study | | |
| *K. pneumoniae* NTUH-K2044 | | Δ*slyA* | | This study | | |
| *K. pneumoniae* NTUH-K2044 | | Δ*uvrY* | | This study | | |
| *K. pneumoniae* NTUH-K2044 | | Δ*waaL* | | This study | | |
| *K. pneumoniae* NTUH-K2044 | | Tn-*wza* | | This study | | |
| *K. pneumoniae* NTUH-K2044 | | Tn-*wza* Δ*waaL* | | This study | | |
| *K. pneumoniae* NTUH-K2044 | | ∆*sapBCDF* Tn-*KP1_3713* | | This study | | |
|  | |  | | |  | |
| **Plasmids** | | | | | | |
| **Name** | **Description** | | | | | **Source** |
| pDS1028 | Transposon delivery vector, Tn*5*-Cm^R^ *ori*6K, pRL27-derived | | | | | (1) |
| pKNG101-Tc | Allelic exchange vector, Tc^R^ | | | | | (16) |
| pFLS5 | pKNG101-Tc derivative, NTUH *waaL* KO, constructed with FS112-FS115 | | | | | This study |
| pFLS6 | pKNG101-Tc derivative, NTUH *wzi* KO, constructed with FS116-FS119 | | | | | This study |
| pFLS7 | pKNG101-Tc derivative, ATCC43816 *arnF* KO, constructed with FS120-FS123 | | | | | This study |
| pFLS8 | pKNG101-Tc derivative, NTUH *argR* KO, constructed with FS132-FS135 | | | | | This study |
| pFLS9 | pKNG101-Tc derivative, NTUH *rfaH* KO, constructed with FS136-FS139 | | | | | This study |
| pFLS10 | pKNG101-Tc derivative, NTUH *slyA* KO, constructed with FS140-FS143 | | | | | This study |
| pFLS11 | pKNG101-Tc derivative, NTUH *uvrY* KO, constructed with FS144-FS147 | | | | | This study |
| pFLS12 | pKNG101-Tc derivative, NTUH/ATCC43816 *emrR*/*mprA* KO, constructed with FS148-FS151 | | | | | This study |
| pFLS13 | pKNG101-Tc derivative, NTUH *ompR* KO, constructed with FS152-FS155 | | | | | This study |
| pFLS14 | pKNG101-Tc derivative, NTUH *arcB* KO, constructed with FS156-FS159 | | | | | This study |
| pFLS16 | pKNG101-Tc derivative, ATCC43816 *rfaH* KO, constructed with FS164-FS167 | | | | | This study |
| pFLS19 | pKNG101-Tc derivative, NTUH *ompR* KO, constructed with FS152-FS155 | | | | | This study |
| pFLS20 | pKNG101-Tc derivative, NTUH *sapBCDF* KO, constructed with FS201,202,204,205 | | | | | This study |
| pFLS22 | pKNG101-Tc derivative, NTUH *mprA* complementation, constructed with FS148/FS150 | | | | | This study |
| pFLS23 | pKNG101-Tc derivative, NTUH *argR* complementation, constructed with FS132/FS134 | | | | | This study |
| pFLS24 | pKNG101-Tc derivative, NTUH *slyA* complementation, constructed with FS140/FS142 | | | | | This study |
| pFLS25 | pKNG101-Tc derivative, NTUH *sapBCDF* complementation, constructed with FS202/FS205 | | | | | This study |
|  | |  | | |  | |
| **Primers** | | | | | | |
| **Name** | **Sequence 5’ – 3’** | | **Description** | | | |
| FS57 | gaccacacgtcgactagtgcnnnnnnnnnnagag | | Random-prime PCR | | | |
| FS58 | gaccacacgtcgactagtgcnnnnnnnnnnacgcc | | Random-prime PCR | | | |
| FS59 | gaccacacgtcgactagtgcnnnnnnnnnngatac | | Random-prime PCR | | | |
| FS60 | gaccacacgtcgactagtgc | | Random-prime PCR | | | |
| FS107 | gagctcgaattcatcgatgatggttgagatgtgta | | TraDIS 5' sequencing primer | | | |
| FS108 | aatgatacggcgaccaccgagatctacaccaggaacacttaacggctgacatgg | | TraDIS 5' PCR primer | | | |
| FS109 | agcctctcaaagcaattttgagtgaca | | transposon-specific primer | | | |
| FS112 | tgtgactagtttgctataagatttaccagcc | | *waaL* KO Fwd | | | |
| FS113 | tgtgactagtcgccattaacccttttacc | | *waaL* KO Rev | | | |
| FS114 | agcgcgattttttgccaaaaagggccgtca | | *waaL* KO overlap-Fwd | | | |
| FS115 | tgacggccctttttggcaaaaaatcgcgct | | *waaL* KO overlap-Rev | | | |
| FS120 | tgtgactagtccaggccggttttgc | | *arnF* KO Fwd | | | |
| FS121 | tgtgactagtatcgcaggattgcacc | | *arnF* KO Rev | | | |
| FS122 | gaccagcgtctgccggggagccggct | | *arnF* KO overlap-Fwd | | | |
| FS123 | agccggctccccggcagacgctggtcccc | | *arnF* KO overlap-Rev | | | |
| FS124 | tgcgaaacgatcctcatcct | | pKNG101 seq | | | |
| FS125 | cccctggatttcactgatgaga | | pKNG101 seq | | | |
| FS132 | tgtgactagtcacattaaacaggtcg | | *argR* KO Fwd | | | |
| FS133 | ccagaggggaattacaagtcacccagattatgg | | *argR* KO overlap-Rev | | | |
| FS134 | tgtgactagtagtgcgcgcttcgat | | *argR* KO Rev | | | |
| FS135 | taatctgggtgacttgtaattcccctctggcta | | *argR* KO overlap-Fwd | | | |
| FS136 | tgtgactagtagcggcgcggcatct | | *rfaH* (NTUH) KO Fwd | | | |
| FS137 | tgacattaacggcgtaatgactcattccacttc | | *rfaH* (NTUH) KO overlap-Rev | | | |
| FS138 | tgtgactagtgacggtagcagctgga | | *rfaH* (NTUH) KO Rev | | | |
| FS139 | gtggaatgagtcattacgccgttaatgtcaa | | *rfaH* (NTUH) KO overlap-Fwd | | | |
| FS140 | tgtgactagttacttccccagctttga | | *slyA* KO Fwd | | | |
| FS141 | ccgggccgcaaagaactccttataattagcttgc | | *slyA* KO overlap-Rev | | | |
| FS142 | tgtgactagttggtaccaggcgcgtt | | *slyA* KO Rev | | | |
| FS143 | agctaattataaggagttctttgcggcccgggct | | *slyA* KO overlap-Fwd | | | |
| FS144 | tgtgactagtcagcctcatcttccatct | | *uvrY* KO Fwd | | | |
| FS145 | cgtcaaaaacatcacaggaataatctccagaatg | | *uvrY* KO overlap-Rev | | | |
| FS146 | tgtgactagtctcttcgaactccaggttc | | *uvrY* KO Rev | | | |
| FS147 | tctggagattattcctgtgatgtttttgacgcaa | | *uvrY* KO overlap-Fwd | | | |
| FS148 | tgtgactagtagcgtcagcagtcactctg | | *mprA* KO Fwd | | | |
| FS149 | tgagcgacgccgcccttgctcaatgggagtaaa | | *mprA* KO overlap-Rev | | | |
| FS150 | tgtgactagtcgctggcgacggtat | | *mprA* KO Rev | | | |
| FS151 | actcccattgagcaagggcggcgtcgctca | | *mprA* KO overlap-Fwd | | | |
| FS152 | tgtgactagtttcgcccgccggcgt | | *ompR* KO Fwd | | | |
| FS153 | aaaagcgcacgcgttaggttcactgcaatagtc | | *ompR* KO overlap-Rev | | | |
| FS154 | tgtgactagtaggcgccatactcgcg | | *ompR* KO Rev | | | |
| FS155 | tattgcagtgaacctaacgcgtgcgcttttcgc | | *ompR* KO overlap-Fwd | | | |
| FS156 | tgtgactagtcccggtttgcgaagag | | *arcB* KO Fwd | | | |
| FS157 | ccggtctgtccggggaggggattccttcacgac | | *arcB* KO overlap-Rev | | | |
| FS158 | tgtgactagtgatcggtcccaatggt | | *arcB* KO Rev | | | |
| FS159 | gtcgtgaaggaatcccctccccggacagaccgg | | *arcB* KO overlap-Fwd | | | |
| FS164 | tgtgactagtcatcctcctgccccat | | *rfaH* (ATCC43816) KO Fwd | | | |
| FS165 | cgacattaacggcgtcaggcagttaactgacgt | | *rfaH* (ATCC43816) KO overlap-Rev | | | |
| FS166 | tgtgactagtaaccggaatttttccacg | | *rfaH* (ATCC43816) KO Rev | | | |
| FS167 | tcagttaactgcctgacgccgttaatgtcg | | *rfaH* (ATCC43816) KO overlap-Fwd | | | |
| FS194 | atgctattaatgccgcgggt | | *wza* qPCR Fwd | | | |
| FS195 | tacctacctcgcccatgaca | | *wza* qPCR Rev | | | |
| FS198 | gcgctttcatgaggcaaagt | | *wcaG* qPCR Fwd | | | |
| FS199 | ctccgcgataaccaaccact | | *wcaG* qPCR Rev | | | |
| FS201 | gccgttgcctgccagtcatggctttttcacctcttcg | | *sapBCDF* KO overlap-Rev | | | |
| FS202 | gtcaactagtgcctgaaagccctgg | | *sapBCDF* KO Rev | | | |
| FS204 | ggtgaaaaagccatgactggcaggcaacggca | | *sapBCDF* KO overlap-Fwd | | | |
| FS205 | gtcaactagtgaggtgagctatctggtggaa | | *sapBCDF* KO Fwd | | | |
| FS297 | gtctcgcgagctttatccga | | *manC* qPCR Fwd | | | |
| FS298 | gtcgggtgatggtttcctgt | | *manC* qPCR Rev | | | |
| FS299 | cggcgacgctgtttgttatc | | *rcsA* qPCR Fwd | | | |
| FS300 | cagattcttccgtacccgca | | *rcsA* qPCR Rev | | | |
| FS303 | ttaaacaggccgaattccag | | *recA* qPCR Fwd | | | |
| FS304 | ccgctttctcaatcagcttc | | *recA* qPCR Rev | | | |
